# Association of image‐defined risk factors with clinical features, histopathology, and outcomes in neuroblastoma

**DOI:** 10.1002/cam4.3663

**Published:** 2020-12-13

**Authors:** William C. Temple, Kieuhoa T. Vo, Katherine K. Matthay, Brunilda Balliu, Christina Coleman, Jennifer Michlitsch, Andrew Phelps, Spencer Behr, Matthew A. Zapala

**Affiliations:** ^1^ Department of Pediatrics UCSF School of Medicine and UCSF Benioff Children's Hospital San Francisco CA USA; ^2^ Department of Biomathematics UCLA Los Angeles CA USA; ^3^ Department of Hematology and Oncology UCSF Benioff Children's Hospital Oakland Oakland, CA USA; ^4^ Department of Radiology and Biomedical Imaging University of California, San Francisco San Francisco CA USA

**Keywords:** pathology, pediatric cancer, surgical oncology, survival

## Abstract

**Background:**

Clinical, molecular, and histopathologic features guide treatment for neuroblastoma, but obtaining tumor tissue may cause complications and is subject to sampling error due to tumor heterogeneity. We hypothesized that image‐defined risk factors (IDRFs) would reflect molecular features, histopathology, and clinical outcomes in neuroblastoma.

**Methods:**

We performed a retrospective cohort study of 76 patients with neuroblastoma or ganglioneuroblastoma. Diagnostic CT scans were reviewed for 20 IDRFs, which were consolidated into five IDRF groups (involvement of multiple body compartments, vascular encasement, tumor infiltration of adjacent organs/structures, airway compression, or intraspinal extension). IDRF groups were analyzed for association with clinical, molecular, and histopathologic features of neuroblastoma.

**Results:**

Patients with more IDRF groups had a higher risk of surgical complications (OR = 3.1, *p* = 0.001). Tumor vascular encasement was associated with increased risk of surgical complications (OR = 5.40, *p* = 0.009) and increased risk of undifferentiated/poorly differentiated histologic grade (OR = 11.11, *p* = 0.013). Tumor infiltration of adjacent organs and structures was associated with decreased survival (HR = 8.90, *p* = 0.007), *MYCN* amplification (OR = 9.91, *p* = 0.001), high MKI (OR = 6.20, *p* = 0.003), and increased risk of International Neuroblastoma Staging System stage 4 disease (OR = 8.96, *p* < 0.001).

**Conclusions:**

The presence of IDRFs at diagnosis was associated with high‐risk clinical, molecular, and histopathologic features of neuroblastoma. The IDRF group tumor infiltration into adjacent organs and structures was associated with decreased survival. Collectively, these findings may assist surgical planning and medical management for neuroblastoma patients.

## Graphical abstract

This is an original research article that explored the association between neuroblastoma image‐defined risk factors (IDRFs) present at diagnosis and various histopathologic, surgical, and survival outcomes. To our knowledge, this is the first report showing that neuroblastoma IDRFs were associated with histopathology in addition to MYCN amplification. It is also the first time, to our knowledge, that specific imaging biomarkers in neuroblastoma, such as tumor vascular encasement and tumor infiltration into adjacent organs and structures, were associated with histopathology and decreased survival.

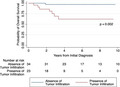

## 1 INTRODUCTION

Neuroblastoma is the most common pediatric extracranial solid tumor, comprising approximately 10%–15% of all pediatric cancer deaths.^1^, ^2^ Over 50% of patients have metastatic disease at diagnosis, and most of these children have poor survival.^3^, ^4^ The behavior of neuroblastoma is remarkably heterogeneous, ranging from spontaneous regression to rapid progression and death, depending on multiple clinical and biologic risk factors.^3^, ^5^ While intensive multimodal therapy will cure only half of children older than 18 months with metastatic disease,^6^ many localized tumors with favorable biology may be cured with surgical resection alone.^7^ This remarkable breadth of outcomes in neuroblastoma makes it imperative to have a reliable and accurate staging and prognostic classification system.

Over the last three decades the most common staging system for neuroblastoma was the International Neuroblastoma Staging System (INSS).^8^, ^9^ INSS staging relies on the extent of surgical resection, which in turn is contingent upon the degree of difficulty resecting the mass. Additionally, definitive resection is often delayed weeks or months to allow for neoadjuvant chemotherapy. Surgical risk factors have been used for over a decade to characterize imaging features that predicted a challenging surgical resection.^10^, ^11^ In the INSS paradigm, there is no method to stage disease before surgery. In 2004, the International Neuroblastoma Risk Group Staging System (INRGSS) developed a pretreatment staging classification based upon image‐defined risk factors (IDRFs) at diagnosis, which has now been adopted by most cooperative clinical trials groups.^9^, ^12^, ^13^

The recent utilization of imaging features coincides with the increasing appreciation for the biologic diversity of neuroblastoma, underlining the importance of obtaining tumor material for determining therapy. The genetic landscape of neuroblastoma exhibits heterogeneity with frequent genomic amplifications and segmental chromosome aberrations.^14^, ^15^, ^16^ These features, combined with gene mutations and histopathology, confer prognostic information as well as potential therapeutic targets.^17^, ^18^, ^19^ However, biopsy or attempted resection of a newly diagnosed neuroblastoma has the potential for bleeding and organ damage, while a needle biopsy may not accurately reflect the total tumor heterogeneity. Employing imaging characteristics has the potential to offer a robust analysis of all tumors, both primary and metastatic, at a single time.^20^, ^21^

Multimodality imaging is an integral part of the work up of a new patient with neuroblastoma, but the amount of information obtained has traditionally been limited to metastatic distribution, tumor location, and tumor volume.^22^ Our group hypothesized that we can apply IDRFs of staging CT scans at diagnosis to assess if there is an association between imaging characteristics and the underlying histologic and genetic aberrations of neuroblastoma, and investigate the possible relationship between IDRFs and clinical outcomes.

## 2 METHODS

### 2.1 Patient selection of the analytic cohort

**FIGURE 1:**
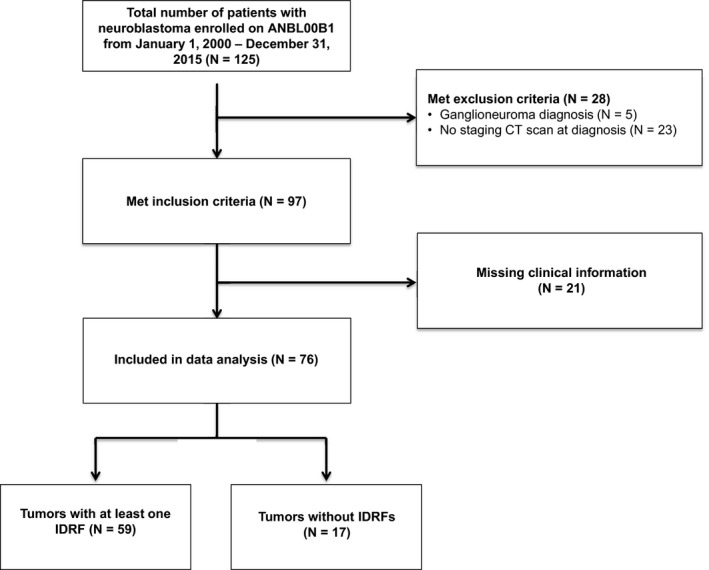
Diagram for patient inclusion

This retrospective cohort study was compliant with HIPAA guidelines. IRB approval was granted for this retrospective study and informed consent was waived. Inclusion criteria in our analytic cohort consisted of patients with a pathologic diagnosis of neuroblastoma or ganglioneuroblastoma who were enrolled on the Children's Oncology Group (COG) biology study, ANBL00B1, from 2000 to 2015 at UCSF Benioff Children's Hospital San Francisco and Oakland, thus, ensuring centralized histopathology and molecular biology. Electronic medical records of 125 patients were reviewed (Figure 1). Forty‐nine patients were excluded, including five with a diagnosis of ganglioneuroma, 23 without an available staging CT scan at diagnosis, and 21 who were missing all essential medical information for the analyses. The final sample size was 76 patients (Figure 1).

### 2.2 Data collection

Clinical, surgical, and pathologic information was obtained from detailed chart review. Electronic medical records (EMR) and available paper charts were reviewed from all patients, including review of available pathology and operative reports. The following information was collected: gender, age at diagnosis, location of primary tumor (abdomen, pelvis, thorax, neck, cervicothoracic, thoracoabdominal, and abdominal‐retroperitoneum‐pelvis), presence of metastases, INSS stage, presence, severity and type of surgical complications, grade of neuroblastic differentiation based on revised International Neuroblastoma Pathology Classification, metaiodobenzylguanidine avidity, MYCN amplification, mitosis‐karyorrhexis index (MKI), DNA ploidy, and clinical outcome (time to death or disease event). Events were defined as disease relapse, progression, death, or development of a second malignancy. INSS was used for staging in this analysis because the INRGSS relies on IDRFs, and because in this patient cohort INSS had been used in the medical record.

### 2.3 Classification of surgical complications

Surgical complications were defined and adapted from criteria by von Allmen et al.^10^ Major intraoperative or postoperative hemorrhage was defined as blood loss greater than 10% of the estimated blood volume (EBV). EBV was calculated using patient's weight at the time of surgery. Renal injury included postoperative development of acute kidney injury or nephrectomy. If primary tumor invaded into the renal capsule and a nephrectomy was performed, this was not considered a surgical complication. Pulmonary complications were defined as development of a pleural effusion requiring chest tube placement or a pneumothorax. If the primary tumor was located in the thoracic cavity, pleural effusions and pneumothoraces were not considered complications. Wound complications were defined as wound dehiscence, abscess formation, or development of cellulitis around the surgical incision site. Vascular injury was defined as any vascular damage intraoperatively that resulted in subsequent clinically significant complications. Some examples of vascular injury included hepatic artery tear with resulting postoperative liver ischemia, thrombosis of vessels in the postoperative period requiring anticoagulation, or sacrifice of major blood vessels. For seven patients, the presence of surgical complications was unable to be determined due to missing clinical data. These patients were excluded from analysis pertaining to surgical complications.

### 2.4 Radiologic analysis

Staging CT scans were reviewed and scored for IDRFs by a board‐certified radiologist with subspecialty certification in pediatric radiology who was blinded to any clinical information and the radiology reports. IDRFs were grouped and divided into five separate binary variable categories for the logistic regression, adapted from Monclair et al^12^: (1) presence of vascular encasement; (2) involvement of multiple body compartments; (3) infiltration of adjacent organs and/or structures; (4) airway compression; and (5) intraspinal tumor extension (Table 1). IDRFs were determined based on the primary tumor and were defined using criteria published by Monclair et al.^12^

**TABLE 1:** Definition of image‐defined risk factor (IDRF) groups, adapted from Monclair et al.^12^ The full list of all individual IDRFs is available from Monclair et al.^12^ Monclair et al analyzed IDRFs by anatomic compartment, while here IDRFs were organized into IDRF groups without regard to anatomic compartment to facilitate radiologic and statistical analysis

IDRF group	Components of each IDRF group
Vascular encasement	Tumor encasing carotid artery, vertebral artery, internal jugular vein, subclavian vessels Tumor encasing the aorta, major aortic vessels^a^, and/or vena cava Tumor encasing iliac vessels
Involvement of multiple body compartments	Tumor involvement into two adjacent body compartments: neck/chest, chest/abdomen, abdomen/pelvis
Infiltration of adjacent organs and/or structures	Tumor infiltration into the porta hepatis, pericardium, diaphragm, kidney, liver, duodeno‐pancreatic block, or mesentery
Airway compression	Tumor compressing the trachea or mainstem bronchi
Intraspinal tumor extension	Tumor extension more than one third into the intraspinal space in the axial plane Tumor involvement in leptomeningeal space Abnormal spinal cord signal, or involvement in the sciatic foramen

^a^ Major aortic vessels include superior mesenteric artery, celiac artery, and iliac vessels.

### 2.5 Statistical analysis

Spearman rank correlations were performed between and within the statistical outcomes and the IDRFs to assess collinearity prior to performing regressions. If two outcomes or IDRFs were highly correlated, only one was selected, to avoid multiple testing burden for variables with similar amount of information as well as potential collinearity problems.

The association between each of the statistical outcome variables was modeled, hereafter referred to as “characteristics” (including clinical, surgical, and histopathologic features of neuroblastoma) and the number of IDRFs (predictor) was modeled using a separate logistic regression for each characteristic and adjusting for age:

(1) $$P\left(Y=1\right)={\text{logit}}^{‐1}\left(\alpha_{0}+\alpha_{1}\text{Age}+\beta \;\text{Number}\;\text{of}\;\text{IDRFs}\right).$$

For the survival characteristic time to event, the following Cox proportional hazard model was used:

$$\log\left(h\left(t\right)\right)=\log\left(h_{0}\left(t\right)\right)+\alpha_{1}\text{Age}+\beta \;\text{Number}\;\text{of}\;\text{IDRFs}),$$

where *β* represent the log(Hazard Ratio) for each unit increase in the number of IDRFs, a positive coefficient indicates worse survival and a negative coefficient indicates better survival for the variable in question.

The association between each of the characteristics and the number of IDRFs was tested:

$$H_{0}:\beta =0\;\text{vs}.\;H_{1}:\beta \neq 0,$$

using a Likelihood Ratio Test (LRT). The false discovery rate (FDR) across characteristics was controlled using the Benjamini–Hochberg (BH) method. The association was modeled between each of the characteristics, except survival, and each of the IDRFs using a logistic regression and adjusting for age:

(2) $$P\left(Y=1\right)={\text{logit}}^{‐1}\left(\alpha_{0}+\alpha_{1}\text{Age}+\beta \;\text{IDRF}\right).$$

For the survival characteristic time to event, the following Cox proportional hazard model was used:

$$\log\left(h\left(t\right)\right)=\log\left(h_{0}\left(t\right)\right)+\alpha_{1}\text{Age}+\beta \;\text{IDRF}).$$

The association between each of the characteristics and each of the IDRFs was tested:

$$H_{0}:\beta =0\;\text{vs}.\;H_{1}:\beta \neq 0,$$

using a LRT.

The FDR across characteristics and IDRFs was controlled using the hierarchical procedure described in Peterson et al.^23^ That is, the characteristics significantly associated with at least one IDRF were identified using the Simes method to obtain p‐values for each characteristic and controlling the FDR at 5% using the BH procedure.

Then, the BH was applied within each characteristic to identify IDRFs significantly associated with each of the significant characteristics from the first step, with the appropriate adjustment for the selection bias introduced in step 1. To control FDR at 5%, the BH‐adjusted *p*‐values within each characteristic need to be smaller than 5% × number of significant characteristics/number of characteristics tested. Since all characteristics were significant in step one, the BH‐adjusted *p*‐value threshold is 5%. The BH‐adjusted *p*‐values are listed in Table 2.

**TABLE 2:** Association of specific image‐defined risk factor (IDRF) groups with clinical, surgical, and histopathologic features of neuroblastoma. To explore associations between an IDRF group (predictor) and clinical, surgical, or histopathologic features of neuroblastoma (outcome), a separate logistic regression was performed for each combination of IDRF group and feature of neuroblastoma, controlling for patient age (Model 2). The false discovery rate across all characteristics and IDRFs tested was controlled using the multi‐outcome hierarchical FDR procedure described by Peterson et al.^23^ Survival analysis was performed as a Cox Proportional Hazard regression model with time‐to‐event endpoint controlling for age to calculate the hazard ratio for survival. Shown are odds/hazard ratio estimates and nominal p‐values for all significant associations

Characteristic	Predictor	Odds ratio/hazard ratio	95% Confidence interval	*p*‐value
Surgical complications	Vascular encasement	5.40	1.65–21.72	0.009
Undifferentiated/poorly differentiated grade	Vascular encasement	11.11	1.98–103.5	0.013
Survival	Tumor infiltration	8.90	1.83–43.32	0.007
INSS stage 4	Tumor infiltration	8.96	2.99–30.29	<0.001
*MYCN* amplification	Tumor infiltration	9.91	2.59–46.59	0.001
High MKI	Tumor infiltration	6.20	1.95–21.29	0.003

In addition, a Kaplan–Meier analysis was performed with the log rank test of overall survival for the presence versus absence of IDRFs (Figure S1) and for the presence or absence of tumor infiltration (Figure 3). Overall survival was defined as the time in days from diagnosis to death or last clinical follow up. All statistical tests were performed in R (version 3.4.0) except for the Kaplan–Meier analysis which was performed in STATA.

## 3 RESULTS

### 3.1 Patient characteristics

The clinical and biological features of the 76 eligible patients are shown in Table 3, with the number of patients available in each category and the percent with IDRFs.

**TABLE 3:** Patient characteristics

Characteristic	Total number of patients with each characteristic who have at least one IDRF		Percentage of patients with each characteristic who have at least one IDRF (%)
Age ≥547 days (N = 37)	N = 28	75.7	
Age <547 days (N = 39)	N = 31	79.5	
Female sex (N = 37)	N = 32	86.5	
Male sex (N = 39)	N = 27	69.2	
Primary tumor site			
Abdomen (N = 46)	N = 38	82.6	
Neck (N = 2)	N = 2	100	
Thorax (N = 12)	N = 4	33.3	
Pelvis (N = 1)	N = 0	0	
Cervicothoracic (N = 3)	N = 3	100	
Thoracoabdominal (N = 2)	N = 2	100	
Abdomen‐pelvis^a^ (N = 10)	N = 10	100	
INSS stage 4 (N = 30)	N = 26	86.7	
Non‐INSS stage 4^b^ (N = 46)	N = 33	71.7	
Surgical complications (N = 30)^b^	N = 30	100	
Absence of surgical complications (N = 36)	N = 20	55.6	
Unknown^c^ (N = 10)			
Gross total resection (N = 54)	N = 41	75.9	
Partial resection (N = 12)	N = 10	83.3	
Unknown^c^ (N = 10)			
Grade of differentiation			
Undifferentiated or poorly differentiated (N = 62)	N = 49	79.0	
Differentiating (N = 8)	N = 4	50.0	
Unknown (N = 6)			
MIBG avidity (N = 62)	N = 48	77.4	
MIBG non‐avid (N = 6)	N = 6	100	
Unknown (N = 8)			
MYCN amplified (N = 15)	N = 14	93.3	
MYCN nonamplified (N = 52)	N = 39	75.0	
Unknown (N = 9)			
High MKI (N = 19)	N = 16	84.2	
Low/intermediate MKI (N = 48)	N = 35	72.9	
Unknown (N = 9)			

Abbreviations: IDRF, image‐defined risk factor; INSS, International Neuroblastoma Staging System; MIBG, metaiodobenzylguanidine

^a^ Abdomen‐pelvis includes tumors with primary location in the abdomen, retroperitoneum, or pelvis.

^b^ Non‐INSS stage 4 disease includes stage 1, 2a, 2b, 3, and 4S.

^c^ Ten patients either did not have surgery, or there is insufficient data available to determine if surgery was performed.

### 3.2 Assessment of collinearity among variables included in analysis

**FIGURE 2:**
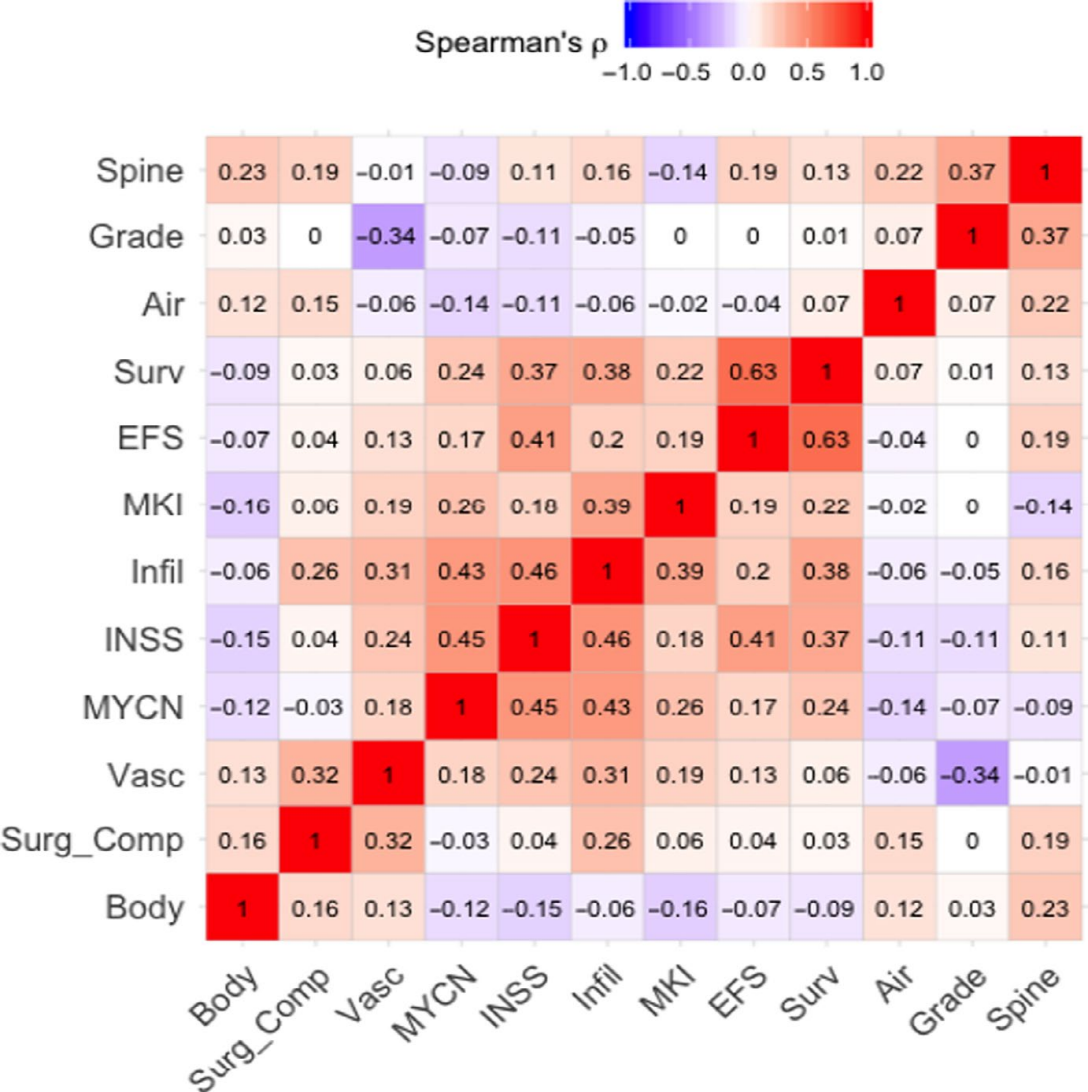
A Spearman Rank correlation matrix was created between all characteristics and image‐defined risk factor (IDRF) groups to determine if collinearity was present. Survival and EFS were moderately correlated (Spearman's *ρ* = 0.63). Additionally, EFS information is missing for three patients, so EFS was excluded from further analysis. Spine, intraspinal extension; Grade, differentiating histologic grade; Air, airway compression; Surv, survival; EFS, event‐free Survival; MKI, mitosis‐karyorrhexis index; Infil, tumor infiltration into adjacent organs and structures; INSS, international neuroblastoma staging system stage 4; MYCN, MYCN amplification; Vasc, tumor vascular encasement; Surg_Comp, surgical complications; Body, tumor involvement of multiple body compartments

To exclude variables that demonstrated collinearity, a Spearman Rank correlation matrix was performed between all characteristics and IDRF groups (Figure 2). Death and disease events were moderately correlated (Spearman's *ρ* = 0.63). Additionally, disease event information was missing for three patients, so it was excluded from further analyses. Overall, there was not a significant degree of collinearity among the remainder of variables included in the study (Figure 2).

### 3.3 Association of IDRF groups with clinical and histopathologic characteristics in neuroblastoma

**FIGURE 3:**
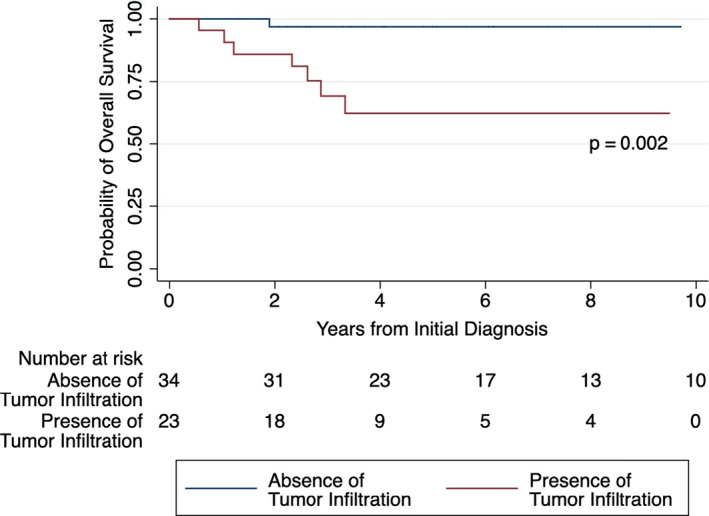
Kaplan–Meier curves for 57 patients, stratified by the presence or absence of tumor infiltration. At 5 years, the overall survival (OS) in the presence of tumor infiltration was 62% (95% CI, 36%–80%), and the OS in the absence of tumor infiltration was 97% (95% CI, 80%–99%)

Association testing was performed between the different clinical and biologic characteristics, and also between these characteristics and each IDRF. All clinical, molecular, and histopathologic features were associated with at least one IDRF group (Tables 2 and 4). Patients with more IDRF groups had a higher risk of surgical complications (OR = 3.1, *p* = 0.001; Table 4). Regarding specific IDRF groups, tumor vascular encasement was associated with increased risk of surgical complications (OR = 5.40, *p* = 0.009; Table 2) and increased risk of an undifferentiated/poorly differentiated histologic grade (OR = 11.11, *p* = 0.013; Table 2). Tumor infiltration of adjacent organs and structures was associated with decreased survival (HR = 8.90, *p* = 0.007; Table 2), increased risk of INSS stage 4 disease (OR = 8.96, *p* < 0.001; Table 2), *MYCN* amplification (OR = 9.91, *p* = 0.001; Table 2), and high MKI (OR = 6.20, *p* = 0.003; Table 2). The 5‐year overall survival was increased in patients who did not have tumor infiltration into adjacent organs/structures, compared to patients who had tumor infiltration (*p* = 0.002; Figure 3). However, patients whose tumors did not have IDRFs did not have a significantly better survival than those who had one more IDRFs (*p* = 0.34; Figure S1).

**TABLE 4:** The number of image‐defined risk factor (IDRF) groups is associated with various clinical, surgical, and histopathologic features of neuroblastoma. To explore associations between the number of IDRF groups and each clinical, surgical, and histopathologic feature of neuroblastoma (characteristic), univariate logistic regression was performed for each characteristic with the number of IDRF groups as predictor, adjusting for age (Model 1), and controlling for multiple comparisons across features using the Benjamini–Hochberg procedure. Survival analysis was performed as a Cox Proportional Hazard regression model with time‐to‐event endpoint controlling for age to calculate the hazard ratio for survival. Shown are the odds/hazard ratios and adjusted *p*‐values

Characteristic	Odds ratio/hazard ratio	95% Confidence interval	Adjusted *p*‐value
Survival	2.03	1.01–4.05	0.092
INSS stage 4	1.85	1.11–3.27	0.051
Surgical complications	3.10	1.6–6.7	0.001
Undifferentiated/poorly differentiated grade	0.73	0.29–1.64	0.454
*MYCN* amplification	1.81	0.92–3.86	0.132
High MKI	1.47	0.85–2.63	0.197

## 4 DISCUSSION

Our work shows that the presence and number of IDRFs were associated with various important clinical, surgical, and histopathologic characteristics of neuroblastoma, and that certain radiologic features present at diagnosis serve as imaging biomarkers to predict tumor behavior. Our findings build on the work of Brisse and colleagues.^24^ Their work specifically analyzed anatomic location of tumors along the sympathetic chain and tumor volume, and how these features correlated with genomic aberrations, *MYCN* amplification, and survival. Brisse et al found that the presence of IDRFs was associated with *MYCN* amplification. Our group validated this and further determined that the presence of IDRFs was associated with the grade of neuroblastic differentiation and high MKI. Interestingly, when each individual group of IDRFs was analyzed, we found that tumor infiltration into adjacent organs or structures was associated with *MYCN* amplification and high MKI, and that tumor vascular encasement was associated with grade of neuroblastic differentiation. To our knowledge, this is the first report showing that IDRFs are associated with histopathology in addition to *MYCN* amplification. It is also the first time, to our knowledge, that specific imaging biomarkers in neuroblastoma, such as tumor vascular encasement and tumor infiltration of adjacent organs or structures, were associated with histopathology.

Additionally, we demonstrated that a specific IDRF group, tumor infiltration into adjacent organs and/or structures at diagnosis, is associated with decreased survival. However, we did not find that the presence of any IDRF is associated with survival, which is consistent with the work of Brisse et al^24^ and Phelps et al.^25^ One of the ultimate goals of using noninvasive imaging biomarkers in neuroblastoma is to achieve prognostic information and de‐escalate therapies that may have excessive toxicity.^26^

Future studies could build on the findings of Avanzini et al^27^ and Irtan et al^28^ to further elucidate how IDRF evolution over the course of treatment is associated with overall survival and event‐free survival. Other studies might analyze the disappearance of IDRFs over the course of disease as a proxy for disease response. Phelps et al^25^ analyzed “IDRF decay” over the course of eight chemotherapy cycles, and determined that there was an overall decrease in the number of IDRFs. They also found that there were different rates of IDRF decrease among *MYCN*‐amplified versus nonamplified tumors. Yoneda et al^29^ investigated how IDRFs within a tumor can change over the course of neoadjuvant chemotherapy. Further studies are needed to better elucidate how changes in IDRF number over the entire treatment course may be clinically relevant. Future studies could also investigate which organs are infiltrated by tumor in order to determine which structures are associated with decreased survival and histopathology such as *MYCN* amplification and high MKI.

A robust body of literature suggests that IDRFs are associated with surgical complications.^11^, ^24^, ^25^, ^30^, ^31^, ^32^ We expanded this understanding by showing that the number of IDRF groups is also associated with surgical complications, and that the presence of vascular encasement at diagnosis is associated with surgical complications. Future studies could further explore which specific surgical complications occur depending upon which IDRF group is present.

Certain features of our study design limit the generalizability of our work. We decided to cluster IDRFs into five groups, and this method of consolidating IDRFs may make direct comparison with other investigations challenging. In addition, a single pediatric radiologist scored the IDRFs. We also had a relatively low sample size, with patients from only two institutions. Numerous patients had some missing data, including clinical, surgical, or histopathologic features, which limit the conclusions of this study. Patients were more likely to have missing data if they were diagnosed closer to the year 2000. This is probably because paper charts were more likely to be used in the early 2000s, with some paper chart information being lost or not recorded in the electronic medical record. It is possible that patients with missing information who were diagnosed in the early 2000s were more likely to have worse outcomes because neuroblastoma treatment evolved significantly during the 15‐year period from the year 2000–2015. Furthermore, differences in surgical expertise may limit the generalizability of relationships to surgical complications and to survival. IDRFs are also used to define INRGSS stage, so using IDRFs to potentially influence treatment decisions will be confounded with INRGSS. Future studies should compare the performance of MRI and CT scans in determining the presence of IDRF groups at diagnosis. We anticipate similar results between CT and MRI scans, as both are recommended for initial staging of neuroblastoma. Based on the work by Sarioglu et al, MRI was superior for detecting intraspinal extension, involvement of multiple body compartments, and metastatic disease, while CT scans were superior at detecting vascular involvement.^33^ Another limitation was that we only controlled for one clinical characteristic, age, because the other characteristics were statistical outcomes. The main point of this study was to assess if preoperative imaging was associated with clinical and biological characteristics, and thus, this could not be adjusted. We acknowledge that other factors, such as institution or specific surgeon, could also confound the results.

Pathology is traditionally paramount in terms of providing information about tumors, but with utilizing additional imaging characteristics there is potential for clinicians to have unfiltered access to neuroblastoma tumor biology in a noninvasive fashion.^21^ With the numerous advances in imaging techniques, this technology helps provide a new intersection between molecular oncology, precision medicine, and radiology.^20^ Histological markers, as well as genetic and genomic anomalies in neuroblastoma, may change the imaging characteristics of neuroblastoma. In this manner, employing imaging information and their association with clinical and histopathologic features of neuroblastoma offers the potential to obtain prognostic information at diagnosis. It is clear further studies are indicated to elucidate how imaging information may predict tumor behavior and response to treatment.

In conclusion, the presence of IDRFs is predictive of surgical complications and correlates with decreased survival and high‐risk histologic features. If imaging principles that incorporate IDRFs and other valuable radiologic features of tumor behavior can be successfully applied to neuroblastoma, a “personalized medicine” approach could be employed using histology, genetics, imaging, and clinical data to cater early treatment to each patient's individual tumor.

## CONFLICT OF INTEREST

The authors made no disclosures.

## AUTHOR CONTRIBUTIONS

William C. Temple: conceptualization, data curation, investigation, methodology, writing ‐ original draft, writing ‐ review and editing. Kieuhoa T. Vo: conceptualization, statistical analysis, investigation, methodology, supervision, writing ‐ review and editing. Katherine K. Matthay: conceptualization, investigation, methodology, supervision, writing ‐ review and editing. Brunilda Balliu: statistical analysis, methodology, writing ‐ review and editing. Christina Coleman: data curation. Jennifer Michlitsch: supervision, writing ‐ review and editing. Andrew Phelps: conceptualization, supervision. Spencer Behr: conceptualization, supervision. Matthew A. Zapala: conceptualization, statistical analysis, investigation, methodology, supervision, writing ‐ review and editing.

## Funding information

Neuroblastoma Research Fund, the Mildred V. Strouss Endowed Chair in Translational Research in Pediatric Oncology.

## Supporting information

Fig S1

## Data Availability

The data that support the findings of this study are available on request from the corresponding author. The data are not publicly available due to privacy or ethical restrictions.

## References

[1] Brodeur GM . Neuroblastoma: biological insights into a clinical enigma. Nat Rev Cancer. 2003;3(3):203‐216. 12612655 10.1038/nrc1014

[2] Irwin MS , Park JR . Neuroblastoma: paradigm for precision medicine. Pediatr Clin North Am. 2015;62(1):225‐256. 25435121 10.1016/j.pcl.2014.09.015

[3] Matthay KK , Maris JM , Schleiermacher G , et al. Neuroblastoma. Nat Rev Dis Primers. 2016;2:16078. 27830764 10.1038/nrdp.2016.78

[4] Ward E , DeSantis C , Robbins A , Kohler B , Jemal A . Childhood and adolescent cancer statistics, 2014. CA Cancer J Clin. 2014;64(2):83‐103. 24488779 10.3322/caac.21219

[5] Maris JM . Recent advances in neuroblastoma. N Engl J Med. 2010;362(23):2202‐2211. 20558371 10.1056/NEJMra0804577 PMC3306838

[6] Pinto NR , Applebaum MA , Volchenboum SL , et al. Advances in risk classification and treatment strategies for neuroblastoma. J Clin Oncol. 2015;33(27):3008‐3017. 26304901 10.1200/JCO.2014.59.4648 PMC4567703

[7] Yao W , Li K , Dong K , Zheng S , Xiao X . Long‐term prognosis of low‐risk neuroblastoma treated by surgery alone: an experience from a single institution of china. World J Pediatr. 2019;15(2):148‐152. 30446974 10.1007/s12519-018-0205-z

[8] Brodeur GM , Pritchard J , Berthold F , et al. Revisions of the international criteria for neuroblastoma diagnosis, staging, and response to treatment. J Clin Oncol. 1993;11(8):1466‐1477. 8336186 10.1200/JCO.1993.11.8.1466

[9] Brisse HJ , McCarville MB , Granata C , et al. Guidelines for imaging and staging of neuroblastic tumors: consensus report from the international neuroblastoma risk group project. Radiology. 2011;261(1):243‐257. 21586679 10.1148/radiol.11101352

[10] von Allmen D , Davidoff AM , London WB , et al. Impact of extent of resection on local control and survival in patients from the COG A3973 study with high‐risk neuroblastoma. J Clin Oncol. 2017;35(2):208‐216. 27870572 10.1200/JCO.2016.67.2642 PMC5455676

[11] Cecchetto G , Mosseri V , De Bernardi B , et al. Surgical risk factors in primary surgery for localized neuroblastoma: the LNESG1 study of the European International Society of Pediatric Oncology Neuroblastoma Group. J Clin Oncol. 2005;23(33):8483‐8489. 16293878 10.1200/JCO.2005.02.4661

[12] Monclair T , Brodeur GM , Ambros PF , et al. The international neuroblastoma risk group (INRG) staging system: an INRG task force report. J Clin Oncol. 2009;27(2):298‐303. 19047290 10.1200/JCO.2008.16.6876 PMC2650389

[13] Cohn SL , Pearson ADJ , London WB , et al. The international neuroblastoma risk group (INRG) classification system: an INRG task force report. J Clin Oncol. 2009;27(2):289‐297. 19047291 10.1200/JCO.2008.16.6785 PMC2650388

[14] Depuydt P , Boeva V , Hocking TD , et al. Genomic amplifications and distal 6q loss: novel markers for poor survival in high‐risk neuroblastoma patients. J Natl Cancer Inst. 2018;110(10):1084‐1093. 29514301 10.1093/jnci/djy022 PMC6186524

[15] Bosse KR , Maris JM . Advances in the translational genomics of neuroblastoma: from improving risk stratification and revealing novel biology to identifying actionable genomic alterations. Cancer. 2016;122(1):20‐33. 26539795 10.1002/cncr.29706 PMC4707066

[16] Deyell RJ , Attiyeh EF . Advances in the understanding of constitutional and somatic genomic alterations in neuroblastoma. Cancer Genet. 2011;204(3):113‐121. 21504710 10.1016/j.cancergen.2011.03.001

[17] Schleiermacher G , Michon J , Ribeiro A , et al. Segmental chromosomal alterations lead to a higher risk of relapse in infants with MYCN‐non‐amplified localised unresectable/disseminated neuroblastoma (a SIOPEN collaborative study). Br J Cancer. 2011;105(12):1940‐1948. 22146831 10.1038/bjc.2011.472 PMC3251887

[18] Thompson D , Vo KT , London WB , et al. Identification of patient subgroups with markedly disparate rates of MYCN amplification in neuroblastoma: a report from the international neuroblastoma risk group project. Cancer. 2016;122(6):935‐945. 26709890 10.1002/cncr.29848 PMC4777644

[19] Attiyeh EF , London WB , Mossé YP , et al. Chromosome 1p and 11q deletions and outcome in neuroblastoma. N Engl J Med. 2005;353(21):2243‐2253. 16306521 10.1056/NEJMoa052399

[20] Jansen RW , van Amstel P , Martens RM , et al. Non‐invasive tumor genotyping using radiogenomic biomarkers, a systematic review and oncology‐wide pathway analysis. Oncotarget. 2018;9(28):20134‐20155. 29732009 10.18632/oncotarget.24893 PMC5929452

[21] Jaffe CC . Imaging and genomics: is there a synergy? Radiology. 2012;264(2):329‐331. 22821693 10.1148/radiol.12120871

[22] Chen AM , Trout AT , Towbin AJ . A review of neuroblastoma image‐defined risk factors on magnetic resonance imaging. Pediatr Radiol. 2018;48(9):1337‐1347. 30078048 10.1007/s00247-018-4117-9

[23] Peterson CB , Bogomolov M , Benjamini Y , Sabatti C . Many phenotypes without many false discoveries: error controlling strategies for multitrait association studies. Genet Epidemiol. 2016;40(1):45‐56. 26626037 10.1002/gepi.21942 PMC4738479

[24] Brisse HJ , Blanc T , Schleiermacher G , et al. Radiogenomics of neuroblastomas: relationships between imaging phenotypes, tumor genomic profile and survival. PLoS One. 2017;12(9):e0185190. 28945781 10.1371/journal.pone.0185190 PMC5612658

[25] Phelps HM , Ndolo JM , Van Arendonk KJ , et al. Association between image‐defined risk factors and neuroblastoma outcomes. J Pediatr Surg. 2019;54(6):1184‐1191. 30885556 10.1016/j.jpedsurg.2019.02.040 PMC6628713

[26] Lucas JT , McCarville MB , Cooper DA , et al. Implications of image‐defined risk factors and primary‐site response on local control and radiation treatment delivery in the management of high‐risk neuroblastoma: is there a role for de‐escalation of adjuvant primary‐site radiation therapy? Int J Radiat Oncol Biol Phys. 2019;103(4):869‐877. 30496881 10.1016/j.ijrobp.2018.11.041 PMC8810202

[27] Avanzini S , Pio L , Erminio G , et al. Image‐defined risk factors in unresectable neuroblastoma: SIOPEN study on incidence, chemotherapy‐induced variation, and impact on surgical outcomes. Pediatr Blood Cancer. 2017;64(11):e26605. 10.1002/pbc.26605 28440012

[28] Irtan S , Brisse HJ , Minard‐Colin V , et al. Image‐defined risk factor assessment of neurogenic tumors after neoadjuvant chemotherapy is useful for predicting intra‐operative risk factors and the completeness of resection. Pediatr Blood Cancer. 2015;62(9):1543‐1549. 25820608 10.1002/pbc.25511

[29] Yoneda A , Nishikawa M , Uehara S , et al. Can neoadjuvant chemotherapy reduce the surgical risks for localized neuroblastoma patients with image‐defined risk factors at the time of diagnosis? Pediatr Surg Int. 2016;32(3):209‐214. 26763000 10.1007/s00383-016-3858-5

[30] Kohler JA , Rubie H , Castel V , et al. Treatment of children over the age of one year with unresectable localised neuroblastoma without MYCN amplification: results of the SIOPEN study. Eur J Cancer. 2013;49(17):3671‐3679. 23907002 10.1016/j.ejca.2013.07.002

[31] Monclair T , Mosseri V , Cecchetto G , De Bernardi B , Michon J , Holmes K . Influence of image‐defined risk factors on the outcome of patients with localised neuroblastoma. A report from the LNESG1 study of the European International Society of Paediatric Oncology Neuroblastoma Group. Pediatr Blood Cancer. 2015;62(9):1536‐1542. 25663103 10.1002/pbc.25460

[32] Pohl A , Erichsen M , Stehr M , et al. Image‐defined risk factors correlate with surgical radicality and local recurrence in patients with neuroblastoma. Klin Padiatr. 2016;228(3):118‐123. 26930233 10.1055/s-0041-111175

[33] Sarioglu FC , Salman M , Guleryuz H , et al. Radiological staging in neuroblastoma: computed tomography or magnetic resonance imaging? Pol J Radiol. 2019;84:46‐53. 10.5114/pjr.2019.82736 PMC6479053 31019594

