# Supplementary material for: Association of image‐defined risk factors with clinical features, histopathology, and outcomes in neuroblastoma
Source: Cancer Med. 2020 Dec 13;10(7):2232–41. doi: 10.1002/cam4.3663 (PMC7982630; doi:10.1002/cam4.3663)
Supplement: Supplementary file 1 — Fig S1 [file CAM4-10-2232-s001.docx]

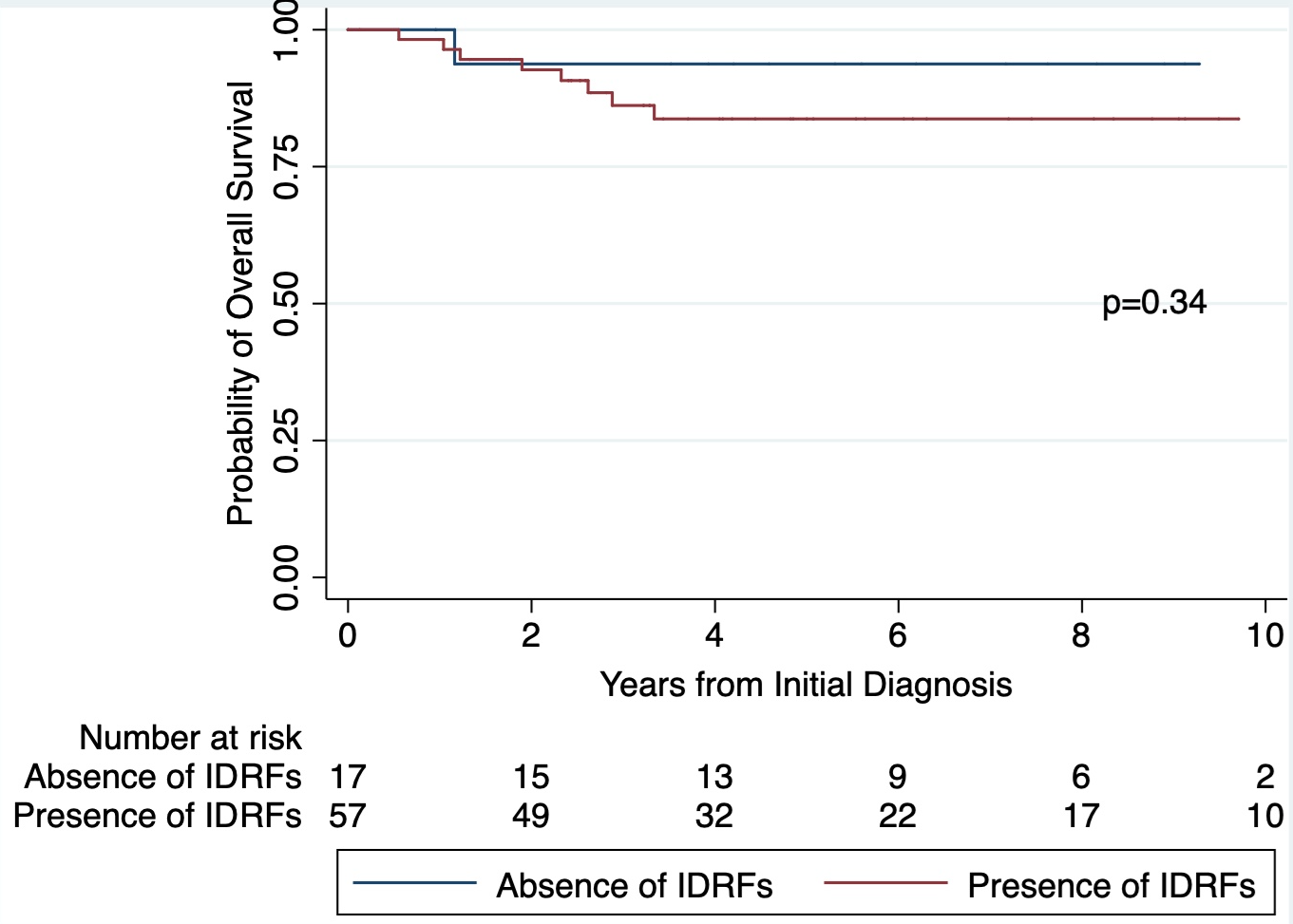


**Supplemental Figure 1. Kaplan-Meier curves for 74 patients, stratified by the presence or absence of image-defined risk factors (IDRFs).** The overall survival (OS) in the presence of IDRFs was 84% (95% CI, 70-92%), and the OS in the absence of IDRFs was 94% (95% CI, 63-99%).
